# A generalization of Fatou’s lemma for extended real-valued functions on *σ*-finite measure spaces: with an application to infinite-horizon optimization in discrete time

**DOI:** 10.1186/s13660-016-1288-5

**Published:** 2017-01-18

**Authors:** Takashi Kamihigashi

**Affiliations:** RIEB, Kobe University, Rokkodai, Nada, Kobe 657-8501 Japan

**Keywords:** Fatou’s lemma, *σ*-finite measure space, infinite-horizon optimization, hyperbolic discounting, existence of optimal paths

## Abstract

Given a sequence $\{f_{n}\}_{n \in \mathbb {N}}$ of measurable functions on a *σ*-finite measure space such that the integral of each $f_{n}$ as well as that of $\limsup_{n \uparrow\infty} f_{n}$ exists in $\overline{\mathbb {R}}$, we provide a sufficient condition for the following inequality to hold:

$$ \limsup_{n \uparrow\infty} \int f_{n} \,d\mu\leq \int\limsup_{n \uparrow\infty} f_{n} \,d\mu. $$

Our condition is considerably weaker than sufficient conditions known in the literature such as uniform integrability (in the case of a finite measure) and equi-integrability. As an application, we obtain a new result on the existence of an optimal path for deterministic infinite-horizon optimization problems in discrete time.

## Introduction

Let $(\Omega, \mathscr {F}, \mu)$ be a measure space. Let $\mathcal {L}(\Omega)$ be the set of measurable functions $f: \Omega\rightarrow\overline{\mathbb {R}}$. A standard version of (reverse) Fatou’s lemma states that given a sequence $\{f_{n}\}_{n \in \mathbb {N}}$ in $\mathcal {L}(\Omega)$, if there exists an integrable function $f \in \mathcal {L}(\Omega)$ such that $f_{n} \leq f$ *μ*-a.e. for all $n \in \mathbb {N}$, then

1.1 $$\begin{aligned} \mathop {\overline {\lim }}_{n \uparrow\infty} \int f_{n} \,d\mu\leq \int \mathop {\overline {\lim }}_{n \uparrow\infty} f_{n} \,d\mu, \end{aligned}$$

where $\mathop {\overline {\lim }}= \limsup$. We call the above inequality the *Fatou inequality*.

Some sufficient conditions for this inequality weaker than the one described above are known. In particular, provided that the integral of each $f_{n}$ as well as that of $\mathop {\overline {\lim }}_{n \uparrow\infty} f_{n}$ exists, ‘uniform integrability’ of $\{f_{n}^{+}\}$ (where $f_{n}^{+}$ is the positive part of $f_{n}$) is a sufficient condition for the Fatou inequality (1.1) in the case of a finite measure (*e.g.*, [1–4]); so is ‘equi-integrability’ of the same sequence in the case of a *σ*-finite measure (see [5, 6]). These conditions are precisely defined in Section 2.

In this paper we provide a sufficient condition for the Fatou inequality (1.1) considerably weaker than the above conditions. Our approach is based on the following assumption, which is maintained throughout the paper.

### Assumption 1.1

$(\Omega, \mathscr {F}, \mu)$ is a *σ*-finite measure space.

Under this assumption there is an increasing sequence of measurable sets of finite measure whose union equals Ω. We use this sequence to specify a ‘direction’ in which we successively approximate the integral of a function.

There is a natural increasing sequence of measurable sets if the measure space is the set of nonnegative integers equipped with the counting measure. In this setting, we provide a simple sufficient condition for the Fatou inequality (1.1) as a corollary of our general result. Applying this condition to a fairly general class of infinite-horizon deterministic optimization problems in discrete time, we establish a new result on the existence of an optimal path. The condition takes a form similar to transversality conditions and other related conditions in dynamic optimization (*e.g.*, [7–10]).

The current line of research was initially motivated by the limitations of the existing applications of Fatou’s lemma to dynamic optimization problems (*e.g.*, [11, 12]). In particular, there are certain cases in which optimal paths exist but the standard version of Fatou’s lemma fails to apply. This is illustrated with some examples following our existence result.

We should mention that there are other important extensions of Fatou’s lemma to more general functions and spaces (*e.g.*, [13–15]). However, to our knowledge, there is no result in the literature that covers our generalization of Fatou’s lemma, which is specific to extended real-valued functions.

In the next section we define the concepts and conditions needed to state our main result and to compare it with some previous results based on uniform integrability and equi-integrability. In Section 3 we state our main result and derive those previous results as consequences. In Section 5 we present two simple examples that cannot be treated by the previous results but that can easily be treated using our result. In Section 6 we show a new result on the existence of an optimal path for infinite-horizon deterministic optimization problems in discrete time. In Section 8 we prove our main result.

## Definitions

Given $f \in \mathcal {L}(\Omega)$, let $f^{+}$ and $f^{-}$ denote the positive and negative parts of *f*, respectively; *i.e.*, $f^{+} = \max\{f, 0\}$ and $f^{-} = \max\{-f, 0\}$. A function $f \in \mathcal {L}(\Omega)$ is called *semi-integrable* if $f^{+}$ or $f^{-}$ is integrable, and *upper* (*lower*) *semi-integrable* if $f^{+}$ ($f^{-}$) is integrable.

We say that a sequence $\{A_{i}\}_{i \in \mathbb {N}}$ in $\mathscr {F}$ is a *σ-finite exhausting sequence* if

2.1 $$\begin{aligned} &\mbox{$\forall $}i \in \mathbb {N}, \quad A_{i} \subset A_{i+1},\qquad \mu(A_{i}) < \infty, \end{aligned}$$

2.2 $$\begin{aligned} & \mu\biggl(\Omega{}\Big\backslash {}\bigcup_{i \in \mathbb {N}} A_{i}\biggr) = 0. \end{aligned}$$

It is easy to see that *μ* is *σ*-finite if and only if there exists a *σ*-finite exhausting sequence. Since we assume that *μ* is *σ*-finite, we have at least one *σ*-finite exhausting sequence.

A sequence $\{f_{n}\}_{n \in \mathbb {N}}$ of integrable functions in $\mathcal {L}(\Omega)$ is called *equi-integrable* (*e.g.*, [6], page 16) if the following conditions hold:

- For any $\epsilon> 0$ there exists $\delta> 0$ such that any $A \in \mathscr {F}$ with $\mu(A) < \delta$ satisfies
  2.3 $$\begin{aligned} \sup_{n \in \mathbb {N}} \int_{A} \vert f_{n}\vert \,d\mu\leq\epsilon. \end{aligned}$$
- For any $\epsilon> 0$ there exists $E \in \mathscr {F}$ with $\mu(E) < \infty$ such that
  2.4 $$\begin{aligned} \sup_{n \in \mathbb {N}} \int_{\Omega\setminus E} \vert f_{n}\vert \,d\mu\leq\epsilon. \end{aligned}$$

Suppose that $\mu(\Omega) < \infty$. A sequence $\{f_{n}\}_{n\in \mathbb {N}}$ of integrable functions in $\mathcal {L}(\Omega)$ is called *uniformly integrable* (*e.g.*, [3], page 144) if

2.5 $$\begin{aligned} \lim_{c \uparrow\infty} \biggl[ \sup_{n \in \mathbb {N}} \int_{\{\vert f_{n}\vert \geq c\}} \vert f_{n}\vert \,d\mu\biggr] = 0. \end{aligned}$$

It is well known that a sequence $\{f_{n}\}_{n \in \mathbb {N}}$ of integrable functions in $\mathcal {L}(\Omega)$ is uniformly integrable if and only if $\sup_{n \in \mathbb {N}} \int \vert f_{n}\vert \,d\mu< \infty$ and condition (a) above holds (*e.g.*, [3], page 144). In the case of a finite measure, condition (b) trivially holds, and thus uniform integrability implies equi-integrability. Conversely, provided that $\sup_{n \in \mathbb {N}} \int \vert f_{n}\vert \,d\mu< \infty$, equi-integrability implies uniform integrability on each measurable set of finite measure; see [6], Proposition 2.8, for related results.

## A generalization of Fatou’s lemma

We are ready to state the main result of this paper.

### Theorem 3.1

*Let* $\{f_{n}\}_{n \in \mathbb {N}}$ *be a sequence of semi*-*integrable functions in* $\mathcal {L}(\Omega)$ *such that* $\mathop {\overline {\lim }}_{n \uparrow\infty} f_{n}$ *is semi*-*integrable*. *Let* $\{B_{i}\}_{i \in \mathbb {N}} \subset \mathscr {F}$ *be a* *σ*-*finite exhausting sequence*. *Suppose that*

3.1 $$\begin{aligned} \mathop {\underline {\lim }}_{i \uparrow\infty} \mathop {\overline {\lim }}_{n \uparrow\infty} \int_{\Omega\setminus A_{i}} f_{n} \,d\mu\leq0 \end{aligned}$$

*for any* *σ*-*finite exhausting sequence* $\{A_{i}\}_{i \in \mathbb {N}} \subset \mathscr {F}$ *such that*

3.2 $$\begin{aligned} \mathrm{(i)} \quad \mbox{$\forall $}i \in \mathbb {N}, \quad A_{i} \subset B_{i},\qquad \mathrm{(ii)}\quad \lim_{i \uparrow\infty} \mu(B_{i} \setminus A_{i}) = 0. \end{aligned}$$

*Then the Fatou inequality* (1.1) *holds*.

### Proof

See Section 8. □

It is shown in the proof (Lemma 8.4) that (2.1) and (3.2) imply (2.2); *i.e.*, (2.1) and (3.2) imply that $\{A_{i}\} _{i \in \mathbb {N}}$ is a *σ*-finite exhausting sequence. Thus in Theorem 3.1, the requirement that $\{A_{i}\}$ be a *σ*-finite exhausting sequence can be replaced with (2.1). However, to verify (3.1) to apply Theorem 3.1, it is useful to have (2.2) instead of deriving it; for example, see the proofs of Corollaries 4.1 and 4.2.

If $\Omega= \mathbb {Z}_{+}$ and *μ* is the counting measure, we obtain a simple sufficient condition for the Fatou inequality:

### Corollary 3.1

*Suppose that* $\Omega= \mathbb {Z}_{+}$ *and that* *μ* *is the counting measure*. *Let* $\{f_{n}\}_{n \in \mathbb {N}}$ *be a sequence of semi*-*integrable functions in* $\mathcal {L}(\Omega)$ *such that* $\mathop {\overline {\lim }}_{n \uparrow\infty} f_{n}$ *is semi*-*integrable*. *Suppose further that*

3.3 $$\begin{aligned} \mathop {\underline {\lim }}_{i \uparrow\infty} \mathop {\overline {\lim }}_{n \uparrow\infty} \sum _{t=i}^{\infty} f_{n}(t) \leq0, \end{aligned}$$

*where the sum is understood as the Lebesgue integral with respect to the counting measure μ*. *Then*

3.4 $$\begin{aligned} \mathop {\overline {\lim }}_{n \uparrow\infty} \sum _{t=0}^{\infty} f_{n}(t) \leq\sum_{t=0}^{\infty} \mathop {\overline {\lim }}_{n \uparrow\infty} f_{n}(t). \end{aligned}$$

### Proof

Assume (3.3). For $i \in \mathbb {N}$, let $B_{i} = \{0, \ldots, i-1\}$. Then $\{B_{i}\}_{i \in \mathbb {N}}$ is a *σ*-finite exhausting sequence. Let $\{A_{i}\}_{i \in \mathbb {N}} \subset \mathscr {F}$ satisfy (3.2). Then $A_{i} = B_{i}$ for sufficiently large *i*. For such *i* we have

3.5 $$\begin{aligned} \sum_{\Omega\setminus A_{i}} f_{n}(t) = \sum _{t=i}^{\infty} f_{n}(t). \end{aligned}$$

Hence (3.1) follows from (3.3). Now (3.4) holds by Theorem 3.1. □

## Known extensions of Fatou’s lemma

The version of Fatou’s lemma stated at the beginning of this paper can be shown as a consequence of Theorem 3.1.

### Corollary 4.1

*Let* $\{f_{n}\}_{n \in \mathbb {N}}$ *be a sequence in* $\mathcal {L}(\Omega)$ *such that for some upper semi*-*integrable function* $f \in \mathcal {L}(\Omega)$ *we have* $f_{n} \leq f$ *μ*-*a*.*e*. *for all* $n \in \mathbb {N}$. *Then the Fatou inequality* (1.1) *holds*.

### Proof

Since $f_{n} \leq f$ *μ*-a.e. for all $n \in \mathbb {N}$ and *f* is upper semi-integrable, $f_{n}$ is upper semi-integrable for each $n \in \mathbb {N}$, and so is $\mathop {\overline {\lim }}_{n \uparrow\infty} f_{n}$. For *any* *σ*-finite exhausting sequence $\{A_{i}\}_{i \in \mathbb {N}}$ we have

4.1 $$\begin{aligned} \mathop {\underline {\lim }}_{i \uparrow\infty} \mathop {\overline {\lim }}_{n \uparrow \infty} \int_{\Omega\setminus A_{i}} f_{n} \,d\mu\leq \mathop {\underline {\lim }}_{i \uparrow \infty} \int_{\Omega\setminus A_{i}} f \,d\mu\leq\lim_{i \uparrow \infty} \int_{\Omega\setminus A_{i}} f^{+} \,d\mu= 0, \end{aligned}$$

where the equality holds by (2.2) since *f* is upper semi-integrable. Now the Fatou inequality (1.1) holds by Theorem 3.1. □

The following version of Fatou’s lemma is shown in [1], page 4, and [2], page 10, and can be derived as a consequence of Theorem 3.1.

### Corollary 4.2

*Suppose that* $\mu(\Omega) < \infty$. *Let* $\{f_{n}\}_{n \in \mathbb {N}}$ *be a sequence of functions in* $\mathcal {L}(\Omega)$ *such that* $\{f_{n}^{+}\}_{n \in \mathbb {N}}$ *is uniformly integrable*. *Suppose further that* $\mathop {\overline {\lim }}_{n \uparrow\infty} f_{n}$ *is semi*-*integrable*. *Then the Fatou inequality* (1.1) *holds*.

### Proof

Recall that uniform integrability of $\{f_{n}^{+}\}$ requires integrability of each $f_{n}^{+}$ and condition (a) in Section 2 with $f_{n}^{+}$ replacing $f_{n}$. Let $\{A_{i}\}_{i \in \mathbb {N}}$ be *any* *σ*-finite exhausting sequence. We have

4.2 $$\begin{aligned} \mathop {\underline {\lim }}_{i \uparrow\infty} \mathop {\overline {\lim }}_{n \in \mathbb {N}} \int_{\Omega \setminus A_{i}} f_{n} \,d\mu\leq\lim_{i \uparrow\infty} \sup_{n \in \mathbb {N}} \int_{\Omega\setminus A_{i}} f_{n}^{+} \,d\mu= 0, \end{aligned}$$

where the equality holds by condition (a) since $\{f_{n}^{+}\}$ is uniformly integrable and $\lim_{i \uparrow\infty} \mu(\Omega \setminus A_{i}) = 0$ by (2.2) and the finiteness of *μ*. Now the Fatou inequality (1.1) holds by Theorem 3.1. □

The next result is a slight variation on the results shown by [5], Lemma 3.3 and [6], Corollary 3.3. The latter results (unlike Corollary 4.3 below) do not require upper semi-integrability of $\mathop {\overline {\lim }}_{n \uparrow\infty} f_{n}$ since they use the upper integral, which always exists, instead of the Lebesgue integral.

### Corollary 4.3

*Let* $\{f_{n}\}_{n \in \mathbb {N}}$ *be a sequence of integrable functions in* $\mathcal {L}(\Omega)$ *such that* $\{f_{n}^{+}\}_{n \in \mathbb {N}}$ *is equi*-*integrable*. *Suppose that* $\mathop {\overline {\lim }}_{n \uparrow\infty} f_{n}$ *is semi*-*integrable*. *Then the Fatou inequality* (1.1) *holds*.

### Proof

By equi-integrability of $\{f_{n}^{+}\}$ and condition (b) in Section 2, there exists a sequence $\{E_{i}\}_{i \in \mathbb {N}}$ in $\mathscr {F}$ such that $\mu(E_{i}) < \infty$ for all $i \in \mathbb {N}$ and

4.3 $$\begin{aligned} \lim_{i \uparrow\infty} \sup_{n \in \mathbb {N}} \int_{\Omega\setminus E_{i}} f_{n}^{+} \,d\mu= 0. \end{aligned}$$

Since *μ* is *σ*-finite, there exists a *σ*-finite exhausting sequence $\{C_{i}\}_{i \in \mathbb {N}}$. For $i \in \mathbb {N}$, let $B_{i} = (\bigcup_{j=1}^{i} E_{j}) \cup C_{i}$. Then $\{B_{i}\}_{i \in \mathbb {N}}$ is also a *σ*-finite exhausting sequence. Let $\{A_{i}\}_{i \in \mathbb {N}}$ be a sequence in $\mathscr {F}$ satisfying (3.2).

Fix $i \in \mathbb {N}$ for the moment. For each $n \in \mathbb {N}$ we have

4.4 $$\begin{aligned} \int_{\Omega\setminus A_{i}} f_{n} \,d\mu& \leq \int_{\Omega \setminus A_{i}} f_{n}^{+} \,d\mu= \int_{\Omega\setminus B_{i}} f_{n}^{+} \,d\mu+ \int_{B_{i} \setminus A_{i}} f_{n}^{+} \,d\mu. \end{aligned}$$

Applying $\sup_{n \in \mathbb {N}}$ to the leftmost and rightmost sides, we obtain

4.5 $$\begin{aligned} \sup_{n \in \mathbb {N}} \int_{\Omega\setminus A_{i}} f_{n} & \leq\sup_{n \in \mathbb {N}} \int_{\Omega\setminus B_{i}} f_{n}^{+} \,d\mu+ \sup _{n \in \mathbb {N}} \int_{B_{i} \setminus A_{i}} f_{n}^{+} \,d\mu. \end{aligned}$$

The first supremum on the right-hand side converges to zero as $i \uparrow\infty$ by (4.3) since $E_{i} \subset B_{i}$ for all $i \in \mathbb {N}$. The second supremum also converges to zero as $i \uparrow\infty$ by (3.2)(ii) and condition (a) in Section 2. It follows that (3.1) holds for any sequence $\{A_{i}\}_{i \in \mathbb {N}}$ in $\mathscr {F}$ satisfying (3.2); thus by Theorem 3.1, the Fatou inequality (1.1) holds. □

## Examples

In each of the examples below, Ω is taken to be an interval in $\mathbb {R}$. Accordingly, $\mathscr {F}$ is taken to be the *σ*-algebra of Lebesgue measurable subsets of Ω, and *μ* the Lebesgue measure restricted to $\mathscr {F}$.

Our first example shows that Theorem 3.1 is a strict generalization of Corollaries 4.2 and 4.3 even in the case of a finite measure.

### Example 5.1

Let $\Omega= [-1, 1] \setminus\{0\}$. For $n \in \mathbb {N}$, define $f_{n} : \Omega\rightarrow \mathbb {R}$ by

5.1 $$\begin{aligned} f_{n}(\omega) = \textstyle\begin{cases} 0 & \text{if $\omega\in[-1, -1/n)$,} \\ -n & \text{if $\omega\in[-1/n, 0)$,} \\ n & \text{if $\omega\in(0, 1/n]$,} \\ 0 & \text{if $\omega\in(1/n, 1]$.} \end{cases}\displaystyle \end{aligned}$$

It is easy to see that there is no upper semi-integrable function that dominates $\{f_{n}\}_{n \in \mathbb {N}}$; thus Corollary 4.1 does not apply. Furthermore, $\{f_{n}^{+}\}$ is not uniformly integrable; indeed, for any $c \geq0$ we have

5.2 $$\begin{aligned} \sup_{n \in \mathbb {N}} \int_{\{f_{n}^{+} \geq c\}} f_{n}^{+} \,d\mu= \sup _{n \in \mathbb {N}: n \geq c} n/n = 1. \end{aligned}$$

Hence Corollary 4.2, which requires uniform integrability of $\{f_{n}^{+}\}$, does not apply either. Neither does Corollary 4.3 since equi-integrability implies uniform integrability on a finite measure space provided that $\sup_{n \in \mathbb {N}} \int \vert f_{n}\vert \,d\mu< \infty$, which is the case here.

By contrast, Theorem 3.1 easily applies. To see this, note that, for each $n \in \mathbb {N}$, $f_{n}$ is integrable, and so is $\lim_{n \uparrow\infty} f_{n}$. For $i \in \mathbb {N}$, let

5.3 $$ B_{i} = [-1, -1/i) \cup(1/i,1]. $$

Then $\{B_{i}\}_{i \in \mathbb {N}}$ is a *σ*-finite exhausting sequence. Let $\{A_{i}\}_{i \in \mathbb {N}}$ be *any* sequence in $\mathscr {F}$ satisfying (3.2)(i). For each fixed $i \in \mathbb {N}$, for any $n \geq i$, we have $f_{n} = 0$ on $B_{i}$, and $\int_{\Omega\setminus A_{i}} f_{n} \,d\mu= \int_{\Omega\setminus B_{i}} f_{n} \,d\mu= 0$. Thus the left-hand side of (3.1) is zero. Hence the Fatou inequality (1.1) holds by Theorem 3.1.

In fact $\int f_{n} \,d\mu= 0$ for all $n \in \mathbb {N}$, and $\lim_{n \uparrow\infty} f_{n} = 0$. Thus both sides of the Fatou inequality (1.1) equal zero.

In the next example, *μ* is not finite, and the sequence $\{f_{n}\} _{n \in \mathbb {N}}$ is uniformly bounded from below.

### Example 5.2

Let $\Omega= \mathbb {R}_{+}$. For $n \in \mathbb {N}$, define $f_{n} : \Omega \rightarrow \mathbb {R}$ by

5.4 $$\begin{aligned} f_{n}(\omega) = \textstyle\begin{cases} 0 & \text{if $\omega\in[0, n)$,} \\ n & \text{if $\omega\in[n, n+1)$,} \\ -1 & \text{if $\omega\in[n+1, 2 n+1)$,} \\ 0 & \text{if $\omega\geq2 n+1$.} \end{cases}\displaystyle \end{aligned}$$

It is easy to see that there is no upper semi-integrable function that dominates $\{f_{n}\}_{n \in \mathbb {N}}$; thus Corollary 4.1 does not apply.

For any $\delta\in(0,1)$ we have

5.5 $$\begin{aligned} \int_{[n,n+\delta)} f_{n}^{+} \,d\mu= n \delta\uparrow \infty\quad \text{as $n \uparrow\infty$.} \end{aligned}$$

Thus $\{f_{n}^{+}\}$ does not satisfy condition (a) in Section 2. To consider condition (b), let $E \in \mathscr {F}$ with $\mu(E) < \infty$. Then

5.6 $$\begin{aligned} \mu(E) = \sum_{n \in \mathbb {Z}_{+}} \mu\bigl(E \cap[n,n+1)\bigr) < \infty, \end{aligned}$$

which implies that $\lim_{n \uparrow\infty} \mu(E \cap[n,n+1)) = 0$. It follows that

5.7 $$\begin{aligned} \int_{\Omega\setminus E} f_{n}^{+} \,d\mu= n \bigl(1-\mu \bigl(E \cap[n, n+1)\bigr)\bigr) \rightarrow\infty\quad \text{as $n \uparrow\infty$.} \end{aligned}$$

Hence $\{f_{n}^{+}\}$ does not satisfy condition (b) either. Therefore $\{f_{n}^{+}\}$ is far from being equi-integrable; as a consequence, Corollary 4.3 does not apply.

To see that Theorem 3.1 applies, note that, for each $n \in \mathbb {N}$, $f_{n}$ is integrable for each *n*, and so is $\lim_{n \uparrow \infty} f_{n}$. For $i \in \mathbb {N}$, let $B_{i} = [0, i)$. Then $\{B_{i}\} _{i \in \mathbb {N}}$ is a *σ*-finite exhausting sequence. Take *any* sequence $\{A_{i}\}_{i \in \mathbb {N}}$ in $\mathscr {F}$ satisfying (3.2)(i). Then for each fixed $i \in \mathbb {N}$ we have $\int_{\Omega \setminus A_{i}} f_{n} \,d\mu= 0$ for all $n \geq i$. Thus the left-hand side of (3.1) equals zero. Hence the Fatou inequality (1.1) holds by Theorem 3.1.

In fact, as in the previous example, we have $\int f_{n} \,d\mu= 0$ for all $n \in \mathbb {N}$, and $\lim_{n \uparrow\infty} f_{n} = 0$; thus both sides of the Fatou inequality (1.1) equal zero.

## An application to infinite-horizon optimization in discrete time

In this section we consider a fairly general class of infinite-horizon maximization problems, establishing a new result on the existence of an optimal path using Corollary 3.1. We start with some notation.

For $t \in \mathbb {Z}_{+}$, let $X_{t}$ be a metric space. For $t \in \mathbb {Z}_{+}$, let $\Gamma_{t} : X_{t} \rightarrow X_{t+1}$ be a compact-valued upper hemicontinuous correspondence in the sense that, for each $x \in X_{t}$, $\Gamma_{t}(x)$ is a nonempty compact subset of $X_{t+1}$, and for any convergent sequence $\{x_{n}\}_{n \in \mathbb {N}}$ in $X_{t}$ with limit $x^{*} \in X_{t}$ and any sequence $\{y_{n}\}_{n \in \mathbb {N}}$ with $y_{n} \in\Gamma_{t}(x_{n})$ for all $n \in \mathbb {N}$, there exists a convergent subsequence $\{y_{n_{i}}\}_{i \in \mathbb {N}}$ of $\{ y_{n}\}_{n \in \mathbb {N}}$ with limit $y^{*} \in\Gamma_{t}(x^{*})$; see [16], page 56 and [17], page 564, concerning this definition of upper hemicontinuity. For $t \in \mathbb {Z}_{+}$, let

6.1 $$\begin{aligned} D_{t} = \bigl\{ (x,y) \in X_{t} \times X_{t+1} : y \in\Gamma_{t}(x)\bigr\} . \end{aligned}$$

For $t \in \mathbb {Z}_{+}$, let $r_{t} : D_{t} \rightarrow \mathbb {R}\cup\{-\infty\} $ be an upper semicontinuous function.

Consider the following maximization problem:

6.2 $$\begin{aligned} &\max_{\{x_{t}\}_{t=1}^{\infty}} \sum_{t = 0}^{\infty} r_{t}(x_{t}, x_{t+1}) \end{aligned}$$

6.3 $$\begin{aligned} &\mathrm{s.t.}\quad x_{t+1} \in\Gamma_{t}(x_{t}),\quad \mbox{$\forall $}t \in \mathbb {Z}_{+}, \end{aligned}$$

6.4 $$\begin{aligned} & \text{$x_{0} \in X_{0}$ given.} \end{aligned}$$

We say that a sequence $\{x_{t}\}_{t=1}^{\infty}$ is a *feasible path* (from $x_{0}$) if it satisfies (6.3). We say that a feasible path $\{x^{*}_{t}\} _{t=1}^{\infty}$ is *optimal* (from $x_{0}$) if for any feasible path $\{x_{t}\}_{t=1}^{\infty}$, we have

6.5 $$\begin{aligned} \sum_{t = 0}^{\infty} r_{t}(x_{t}, x_{t+1}) \leq\sum _{t = 0}^{\infty} r_{t}\bigl(x^{*}_{t}, x^{*}_{t+1}\bigr), \end{aligned}$$

where $x^{*}_{0} = x_{0}$. For the above inequality to make sense, we assume the following.

### Assumption 6.1

For each feasible path $\{x_{t}\}_{t=1}^{\infty}$, we have

6.6 $$\begin{aligned} \sum_{t=0}^{\infty} \max\bigl\{ r_{t}(x_{t}, x_{t+1}), 0\bigr\} < \infty. \end{aligned}$$

In other words, the mapping $r_{t}(x_{t}, x_{t+1}): t \mapsto \mathbb {R}\cup\{ -\infty\}$ is upper semi-integrable.

We are ready to show our existence result.

### Proposition 6.1

*Let Assumption* 6.1 *hold*. *Suppose that*, *for any sequence* $\{ \{x_{t}^{n}\}_{t=1}^{\infty}\}_{n \in \mathbb {N}}$ *of feasible paths*, *we have*

6.7 $$\begin{aligned} \mathop {\underline {\lim }}_{i \uparrow\infty} \mathop {\overline {\lim }}_{n \uparrow \infty} \sum _{t=i}^{\infty} r_{t}\bigl(x_{t}^{n}, x_{t+1}^{n}\bigr) \leq0. \end{aligned}$$

*Then there exists an optimal path*.

### Proof

Let

6.8 $$\begin{aligned} \nu= \sup\sum_{t = 0}^{\infty} r_{t}(x_{t}, x_{t+1}), \end{aligned}$$

where the supremum is taken over all feasible paths $\{x_{t}\} _{t=1}^{\infty}$. By the definition of *ν*, there exists a sequence $\{\{x_{t}^{n}\}_{t=1}^{\infty}\}_{n \in \mathbb {N}}$ of feasible paths such that

6.9 $$\begin{aligned} \lim_{n \uparrow\infty} \sum_{t = 0}^{\infty} r_{t}\bigl(x_{t}^{n}, x_{t+1}^{n} \bigr) = \nu. \end{aligned}$$

Since $\Gamma_{0}(x_{0})$ is compact, there exists a convergent subsequence $\{x_{1}^{n_{j}}\}_{j \in \mathbb {N}}$ of $\{x_{1}^{n}\}_{n \in \mathbb {N}}$ with limit $x^{*}_{1} \in\Gamma_{0}(x_{0})$. By the definition of upper hemicontinuity, there exists a convergent subsequence of $\{ x_{2}^{n_{j}}\}_{j \in \mathbb {N}}$ with limit $x^{*}_{2} \in\Gamma _{1}(x^{*}_{1})$. Continuing this way and using the diagonal argument, we see that there exists a subsequence of $\{\{x_{t}^{n}\} _{t=1}^{\infty}\}_{n \in \mathbb {N}}$, again denoted by $\{\{x_{t}^{n}\} _{t=1}^{\infty}\}_{n \in \mathbb {N}}$, such that, for each $t \in \mathbb {N}$, $x_{t}^{n}$ converges to some $x^{*}_{t}$ as $n \uparrow\infty$, and for each $t \in \mathbb {Z}_{+}$, $x^{*}_{t+1} \in\Gamma_{t}(x^{*}_{t})$. Hence $\{x^{*}_{t}\}_{t=1}^{\infty}$ is a feasible path, which implies that

6.10 $$\begin{aligned} \sum_{t=0}^{\infty} r_{t}\bigl(x^{*}_{t}, x^{*}_{t+1} \bigr) \leq\nu. \end{aligned}$$

To apply Corollary 3.1, let $f_{n}(t) = r_{t}(x_{t}^{n}, x_{t+1}^{n})$ for $t \in \mathbb {Z}_{+}$. By Assumption 6.1, for each $n \in \mathbb {N}$, $f_{n}(t)$ is an upper semi-integrable function of $t \in \mathbb {Z}_{+}$. For $t \in \mathbb {Z}_{+}$, let $f^{*}(t) = r_{t}(x^{*}_{t}, x^{*}_{t+1})$. Since $\{x^{*}_{t}\}_{t=1}^{\infty}$ is feasible as shown above, $f^{*}(t)$ is also an upper semi-integrable function of $t \in \mathbb {Z}_{+}$ by Assumption 6.1. For each $t \in \mathbb {Z}_{+}$, by upper semicontinuity of $r_{t}$ we have

6.11 $$\begin{aligned} \mathop {\overline {\lim }}_{n \uparrow\infty} f_{n}(t) = \mathop {\overline {\lim }}_{n \uparrow\infty } r_{t}\bigl(x_{t}^{n}, x_{t+1}^{n}\bigr) \leq r_{t}\bigl(x^{*}_{t}, x^{*}_{t+1}\bigr) = f^{*}(t). \end{aligned}$$

Since the rightmost side is an upper semi-integrable function of $t \in \mathbb {Z}_{+}$, so is the leftmost side. Note that (3.3) directly follows from (6.7). Thus we can apply Corollary 3.1 to obtain (3.4), which is written here as

6.12 $$\begin{aligned} \mathop {\overline {\lim }}_{n \uparrow\infty} \sum_{t=0}^{\infty} r_{t}\bigl(x_{t}^{n}, x_{t+1}^{n} \bigr) \leq\sum_{t=0}^{\infty} \mathop {\overline {\lim }}_{n \uparrow\infty} r_{t}\bigl(x_{t}^{n}, x_{t+1}^{n}\bigr). \end{aligned}$$

We are ready to show that $\{x^{*}_{t}\}_{t=1}^{\infty}$ is an optimal path. Recall from (6.9) that

6.13 $$\begin{aligned} \nu& = \lim_{n \uparrow\infty} \sum _{t=0}^{\infty} r_{t}\bigl(x_{t}^{n}, x_{t+1}^{n}\bigr) \end{aligned}$$

6.14 $$\begin{aligned} & \leq\sum_{t=0}^{\infty} \mathop {\overline {\lim }}_{n \uparrow\infty} r_{t}\bigl(x_{t}^{n}, x_{t+1}^{n}\bigr) \end{aligned}$$

6.15 $$\begin{aligned} & \leq\sum_{t=0}^{\infty} r_{t}\bigl(x^{*}_{t}, x^{*}_{t+1} \bigr), \end{aligned}$$

where (6.14) uses (6.12), and (6.15) uses (6.11). It follows from (6.13)-(6.15) and (6.10) that $\{x^{*}_{t}\}_{t=1}^{\infty}$ is an optimal path. □

As a simple consequence of Proposition 6.1, we obtain a result that can be viewed as an abstract version of the existence result shown in [12], Proposition 4.1; see [18], Theorem 1, for a similar result that requires stronger assumptions.

### Corollary 6.1

*Suppose that there exists an integrable function* $\overline{f} : \mathbb {Z}_{+} \rightarrow \mathbb {R}_{+}$ *such that*, *for any feasible path* $\{x_{t}\} _{t=1}^{\infty}$, *we have*

6.16 $$\begin{aligned} \mbox{$\forall $}t \in \mathbb {Z}_{t}, \quad r_{t}(x_{t}, x_{t+1}) \leq\overline{f}(t). \end{aligned}$$

*Then there exists an optimal path*.

### Proof

Note that (6.16) implies Assumption 6.1. Thus to apply Proposition 6.1, it suffices to verify (6.7) for an arbitrary sequence $\{\{x_{t}^{n}\}_{t=1}^{\infty}\}_{n \in \mathbb {N}}$ of feasible paths. Let $\{\{x_{t}^{n}\}_{t=1}^{\infty}\}_{n \in \mathbb {N}}$ be a sequence of feasible paths. Then by (6.16) we have

6.17 $$\begin{aligned} \mathop {\underline {\lim }}_{i \uparrow\infty} \mathop {\overline {\lim }}_{n \uparrow\infty} \sum _{t=i}^{\infty} r_{t}\bigl(x_{t}^{n}, x_{t+1}^{n}\bigr) \leq \mathop {\underline {\lim }}_{i \uparrow\infty} \mathop {\overline {\lim }}_{n \uparrow\infty} \sum_{t=i}^{\infty} \overline{f}(t) = \mathop {\underline {\lim }}_{i \uparrow\infty} \sum_{t=i}^{\infty} \overline{f}(t) = 0, \end{aligned}$$

where the last equality holds by integrability of *f̅*. It follows that (6.7) holds; hence an optimal path exists by Proposition 6.1. □

Corollary 6.1 can be shown directly by using Fatou’s lemma to conclude (6.12) from (6.16) in the proof of Proposition 6.1. As illustrated in the next section, Proposition 6.1 covers some important cases to which Corollary 6.1 fails to apply.

## Examples of optimization problems

To illustrate the significance of our existence result, we consider two special cases of the following example.

### Example 7.1

Let $u : \mathbb {R}_{+} \rightarrow \mathbb {R}\cup\{-\infty\}$ be a strictly increasing, upper semicontinuous function. Let $\delta: \mathbb {R}_{+} \rightarrow \mathbb {R}_{++}$ be a strictly decreasing function. Consider the following maximization problem:

7.1 $$\begin{aligned} &\max_{\{x_{t}\}_{t=1}^{\infty}} \sum_{t=0}^{\infty} \delta(t) u(c_{t}) \end{aligned}$$

7.2 $$\begin{aligned} &\mathrm{s.t.} \quad c_{t} + x_{t+1} = x_{t},\qquad c_{t}, x_{t+1} \geq0, \quad \mbox{$\forall $}t \in \mathbb {Z}_{+}, \end{aligned}$$

7.3 $$\begin{aligned} & \text{$x_{0} \in \mathbb {R}_{+}$ given.} \end{aligned}$$

In economics, *u* and *δ* are known as a utility function and a discount function, respectively. The above maximization problem is a special case of (6.2)-(6.4) such that, for all $t \in \mathbb {Z}_{+}$, $X_{t} = \mathbb {R}_{+}$ and

7.4 $$\begin{aligned} &r_{t}(x, y) = \delta(t) u(x - y), \end{aligned}$$

7.5 $$\begin{aligned} &\Gamma_{t}(x) = \{y \in \mathbb {R}_{+}: 0 \leq y \leq x\}. \end{aligned}$$

It is easy to see from (7.2) that

7.6 $$\begin{aligned} \mbox{$\forall $}t \in \mathbb {Z}_{+}, \quad c_{t}, x_{t} \leq x_{0}. \end{aligned}$$

For simplicity, we assume that there exists $\theta> 0$ such that

7.7 $$\begin{aligned} \text{(i)}\quad \mbox{$\forall $}c \geq0,\quad u(c) \leq\theta c,\qquad \text{(ii)}\quad u(x_{0}) > 0. \end{aligned}$$

(Condition (ii) above does not depend on *θ*.) It is easy to see that condition (i) above implies Assumption 6.1; see (7.13)-(7.16) for details.

### Example 7.2

Consider Example 7.1. Most discrete-time economic models assume an exponential discount function of the form

7.8 $$\begin{aligned} \mbox{$\forall $}t \in \mathbb {Z}_{+}, \quad \delta(t) = \beta^{t} \end{aligned}$$

for some $\beta\in(0,1)$. In this case, Corollary 6.1 easily applies. To see this, let $\overline{f}(t) = \beta^{t} u(x_{0})$ for $t \in \mathbb {Z}_{+}$. Then $\overline{f} : \mathbb {Z}_{+} \rightarrow \mathbb {R}_{+}$ is integrable, and (6.16) holds by (7.6). Hence an optimal path exists by Corollary 6.1.

### Example 7.3

Consider Example 7.1 again. Although exponential discounting (7.8) is technically convenient (implying time consistency), experimental evidence suggests that ‘hyperbolic discounting’ is more plausible; see, *e.g.*, [19], page 1. A simple hyperbolic discount function can be specified as follows:

7.9 $$\begin{aligned} \mbox{$\forall $}t \in \mathbb {Z}_{+},\quad \delta(t) = \frac{1}{1 + \alpha t} \end{aligned}$$

for some $\alpha> 0$.

In this example, Corollary 6.1 does not apply since there exists no integrable function $\overline{f} : \mathbb {Z}_{+} \rightarrow \mathbb {R}_{+}$ satisfying (6.16) for all feasible paths. To see this, define the feasible path $\{\tilde{x}_{t}^{n}\}_{t=1}^{\infty}$ for each $n \in \mathbb {N}$ by

7.10 $$\begin{aligned} \tilde{x}_{t}^{n} = \textstyle\begin{cases} x_{0} & \text{if $t \leq n$,} \\ 0 & \text{if $t \geq n+1$.} \end{cases}\displaystyle \end{aligned}$$

Then

7.11 $$\begin{aligned} r_{t}\bigl(\tilde{x}_{t}^{n}, \tilde{x}_{t+1}^{n}\bigr) = \textstyle\begin{cases} u(x_{0})/(1 + \alpha t) & \text{if $t = n$,} \\ u(0)/(1 + \alpha t) & \text{otherwise.} \end{cases}\displaystyle \end{aligned}$$

Hence any *f̅* satisfying (6.16) must satisfy

7.12 $$\begin{aligned} \overline{f}(t) \geq u(x_{0})/(1+\alpha t),\quad \mbox{$\forall $}t \in \mathbb {Z}_{+}. \end{aligned}$$

Since the right-hand side is not upper semi-integrable in $t \in \mathbb {Z}_{+}$ by (7.7)(ii), there exists no integrable function *f̅* satisfying (6.16) for all feasible paths. Hence Corollary 6.1 does not apply.

However, Proposition 6.1 still applies. To see this, let $\{ \{x_{t}^{n}\}_{t=1}^{\infty}\}_{n \in \mathbb {N}}$ be a sequence of feasible paths. For any $n, i \in \mathbb {N}$ we have

7.13 $$\begin{aligned} \sum_{t=i}^{\infty} r_{t}\bigl(x_{t}^{n}, x_{t+1}^{n} \bigr) & = \sum_{t = i}^{\infty} \frac{u(x_{t}^{n} - x_{t+1}^{n})}{1 + \alpha t} \end{aligned}$$

7.14 $$\begin{aligned} & \leq\sum_{t=i}^{\infty} \frac{\theta(x_{t}^{n} - x_{t+1}^{n})}{1 + \alpha i} \end{aligned}$$

7.15 $$\begin{aligned} & = \frac{\theta\sum_{t=i}^{\infty}(x_{t}^{n} - x_{t+1}^{n})}{1 + \alpha i} \end{aligned}$$

7.16 $$\begin{aligned} & \leq\frac{\theta x_{i}^{n}}{1 + \alpha i} \leq \frac{\theta x_{0}}{1 + \alpha i}, \end{aligned}$$

where (7.14) uses (7.7)(i), and the second inequality in (7.16) uses (7.6). It follows that

7.17 $$\begin{aligned} \mathop {\underline {\lim }}_{i \uparrow\infty} \mathop {\overline {\lim }}_{n \uparrow\infty} \sum _{t=i}^{\infty} r_{t}\bigl(x_{t}^{n}, x_{t+1}^{n}\bigr) \leq \mathop {\underline {\lim }}_{i \uparrow\infty} \frac{\theta x_{0}}{1 + \alpha i} = 0. \end{aligned}$$

Thus (6.7) holds; hence an optimal path exists by Proposition 6.1.

In the above example, the hyperbolic discount function (7.9) is used to show that Corollary 6.1 does not apply. The only property of the discount function required to apply Proposition 6.1 is the equality in (7.17). We summarize this observation in the following example.

### Example 7.4

Consider Example 7.1 again. Suppose that

7.18 $$\begin{aligned} \lim_{t \uparrow\infty} \delta(t) = 0. \end{aligned}$$

Then the argument of Example 7.3 shows that an optimal path exists by Proposition 6.1.

## Proof of Theorem 3.1

### Preliminaries

Throughout the proof, we fix $\{f_{n}\}_{n \in \mathbb {N}}$ and $\{B_{i}\}_{i \in \mathbb {N}}$ to be given by Theorem 3.1. Define $f^{*} = \mathop {\overline {\lim }}_{n \uparrow\infty} f_{n}$. For $n \in \mathbb {N}$, define $\hat {f}_{n} = \sup_{m \geq n} f_{m}$. We have

8.1 $$\begin{aligned} f^{*} = \lim_{n \uparrow\infty} \hat{f}_{n}. \end{aligned}$$

The following observation helps to simplify the proof.

#### Lemma 8.1

*If* $f^{*}$ *is not upper semi*-*integrable*, *then the Fatou inequality* (1.1) *holds*.

#### Proof

Suppose that $f^{*}$ is not upper semi-integrable. Then $\int (f^{*})^{+} \,d\mu= \infty$, and $f^{*}$ must be lower semi-integrable (*i.e.*, $\int(f^{*})^{-} \,d\mu< \infty$) since $f^{*}$ is semi-integrable by hypothesis. It follows that $\int f^{*} \,d\mu= \int(f^{*})^{+} \,d\mu- \int(f^{*})^{-} \,d\mu= \infty$. Thus the Fatou inequality (1.1) trivially holds. □

Since the above result covers the case in which $f^{*}$ is not upper semi-integrable, we assume the following for the rest of the proof.

#### Assumption 8.1

$f^{*}$ is upper semi-integrable.

### Lemmas

We establish three lemmas before completing the proof of Theorem 3.1.

#### Lemma 8.2

*Suppose that there exists a* *σ*-*finite exhausting sequence* $\{ A_{i}\}_{i \in \mathbb {N}}$ *satisfying* (3.1) *and the following*:

8.2 $$\begin{aligned} \mbox{$\forall $}i \in \mathbb {N},\quad \mathop {\overline {\lim }}_{n \uparrow\infty} \int_{A_{i}} f_{n} \,d\mu\leq \int_{A_{i}} f^{*} \,d\mu. \end{aligned}$$

*Then the Fatou inequality* (1.1) *holds*.

#### Proof

Since each $f_{n}$ is semi-integrable, we have

8.3 $$\begin{aligned} \mbox{$\forall $}i, n \in \mathbb {N},\quad \int f_{n} \,d\mu= \int_{A_{i}} f_{n} \,d\mu+ \int_{\Omega\setminus A_{i}} f_{n} \,d\mu. \end{aligned}$$

By (3.1) there exists a subsequence $\{A_{i_{k}}\}_{k \in \mathbb {N}}$ of $\{A_{i}\}_{i \in \mathbb {N}}$ such that

8.4 $$\begin{aligned} &\mbox{$\forall $}k \in \mathbb {N},\quad \mathop {\overline {\lim }}_{n \uparrow\infty} \int_{\Omega\setminus A_{i_{k}}} f_{n} \,d\mu< \infty, \end{aligned}$$

8.5 $$\begin{aligned} & \lim_{k \uparrow\infty} \mathop {\overline {\lim }}_{n \uparrow \infty} \int_{\Omega \setminus A_{i_{k}}} f_{n} \,d\mu\leq0. \end{aligned}$$

Note that $\{A_{i_{k}}\}_{k \in \mathbb {N}}$ is a *σ*-finite exhausting sequence.

Fix $k \in \mathbb {N}$ for the moment. Replacing *i* with $i_{k}$ in (8.3) and applying $\mathop {\overline {\lim }}_{n \uparrow\infty}$ to both sides of the resulting equation, we obtain

8.6 $$\begin{aligned} \mathop {\overline {\lim }}_{n \uparrow\infty} \int f_{n} \,d\mu& = \mathop {\overline {\lim }}_{n \uparrow\infty} \biggl[ \int_{A_{i_{k}}} f_{n} \,d\mu+ \int_{\Omega\setminus A_{i_{k}}} f_{n} \,d\mu\biggr] \end{aligned}$$

8.7 $$\begin{aligned} & \leq \mathop {\overline {\lim }}_{n \uparrow\infty} \int_{A_{i_{k}}} f_{n} \,d\mu+ \mathop {\overline {\lim }}_{n \uparrow\infty} \int_{\Omega\setminus A_{i_{k}}} f_{n} \,d\mu \end{aligned}$$

8.8 $$\begin{aligned} & \leq \int_{A_{i_{k}}} f^{*} \,d\mu+ \mathop {\overline {\lim }}_{n \uparrow\infty} \int_{\Omega\setminus A_{i_{k}}} f_{n} \,d\mu, \end{aligned}$$

where (8.7) holds by (8.4), and (8.8) uses (8.2).

Since $f^{*}$ is upper semi-integrable and $\{A_{i_{k}}\}_{k \in \mathbb {N}}$ is a *σ*-finite exhausting sequence, we have $\lim_{k \uparrow \infty} \int_{A_{i_{k}}} f^{*} \,d\mu= \int f^{*} \,d\mu< \infty$. Thus applying $\lim_{k \uparrow\infty}$ to the right-hand side of (8.8) yields

8.9 $$\begin{aligned} \mathop {\overline {\lim }}_{n \uparrow\infty} \int f_{n} \,d\mu\leq \int f^{*} \,d\mu+ \lim_{k \uparrow\infty} \mathop {\overline {\lim }}_{n \uparrow\infty} \int_{\Omega\setminus A_{i_{k}}} f_{n} \,d\mu\leq \int f^{*} \,d\mu, \end{aligned}$$

where the last inequality uses (8.5). The Fatou inequality (1.1) follows. □

#### Lemma 8.3

*Let* $\{A_{i}\}_{i \in \mathbb {N}}$ *be a sequence in* $\mathscr {F}$ *such that*, *for each* $i \in \mathbb {N}$, $\mu(A_{i}) < \infty$ *and* $\hat{f}_{n}^{+}$ *converges to* $(f^{*})^{+}$ *uniformly on* $A_{i}$ *as* $n \uparrow\infty$. *Then* $\{ A_{i}\}_{i \in \mathbb {N}}$ *satisfies* (8.2).

#### Proof

Let $i \in \mathbb {N}$. Let $\delta> 0$. Since $\hat{f}_{n}^{+}$ converges to $(f^{*})^{+}$ uniformly on $A_{i}$ as $n \uparrow\infty$, for sufficiently large $n \in \mathbb {N}$ we have $f_{n} \leq\hat{f}_{n} \leq \hat{f}_{n}^{+} \leq(f^{*})^{+} + \delta$ on $A_{i}$. Since $(f^{*})^{+}$ is integrable by Assumption 8.1 and $\mu (A_{i}) < \infty$, (8.2) holds by Fatou’s lemma. □

#### Lemma 8.4

*Let* $\{A_{i}\}_{i \in \mathbb {N}}$ *be a sequence in* $\mathscr {F}$ *satisfying* (2.1) *and* (3.2). *Then* $\{A_{i}\}$ *is a* *σ*-*finite exhausting sequence*.

#### Proof

Since $\{A_{i}\}$ satisfies (2.1) by hypothesis, it suffices to verify (2.2). For any $i, j \in \mathbb {N}$ with $i \leq j$, by (2.1) for $\{B_{i}\}$, we have

8.10 $$\begin{aligned} \mu(B_{i} \setminus A_{j}) \leq \mu(B_{j} \setminus A_{j}) \rightarrow0 \quad \text{as $j \uparrow \infty$,} \end{aligned}$$

where the convergence holds by (3.2). It follows that

8.11 $$\begin{aligned} \mbox{$\forall $}i \in \mathbb {N},\quad \mu\biggl(B_{i} {}\Big\backslash {} \bigcup _{j \in \mathbb {N}} A_{j}\biggr) = \lim_{j \uparrow\infty} \mu(B_{i} \setminus A_{j}) = 0. \end{aligned}$$

Therefore

8.12 $$\begin{aligned} \mu\biggl(\bigcup_{i \in \mathbb {N}} B_{i} {}\Big\backslash {}\bigcup_{j \in \mathbb {N}} A_{j}\biggr) = \lim_{i \uparrow\infty} \mu\biggl(B_{i} {}\Big\backslash {}\bigcup_{j \in \mathbb {N}} A_{j}\biggr) = 0. \end{aligned}$$

Since $\bigcup_{i \in \mathbb {N}} A_{i} \subset\bigcup_{i \in \mathbb {N}} B_{i}$, we have

8.13 $$\begin{aligned} \mu\biggl(\Omega{}\Big\backslash {}\bigcup_{i \in \mathbb {N}} A_{i}\biggr) & = \mu\biggl(\Omega{}\Big\backslash {}\bigcup _{i \in \mathbb {N}} B_{i}\biggr) + \mu\biggl(\bigcup _{i \in \mathbb {N}} B_{i} {}\Big\backslash {}\bigcup _{i \in \mathbb {N}} A_{i}\biggr) = 0, \end{aligned}$$

where the last equality holds by (2.2) for $\{B_{i}\}$ and (8.12). It follows that $\{A_{i}\}$ satisfies (2.2). □

### Completing the proof of Theorem 3.1

Note from (8.1) that $(f^{*})^{+} = \lim_{n \uparrow \infty} \hat{f}_{n}^{+}$. Let $\{\epsilon_{i}\}_{i \in \mathbb {N}}$ be a sequence in $\mathbb {R}_{++}$ such that $\lim_{i \uparrow\infty} \epsilon _{i} = 0$. For each $i \in \mathbb {N}$, by Egorov’s theorem there exists $E_{i} \in \mathscr {F}$ such that $E_{i} \subset B_{i}$, $\mu(B_{i} \setminus E_{i}) < \epsilon_{i}$, and $\hat{f}_{n}^{+}$ converges to $(f^{*})^{+}$ uniformly on $E_{i}$ as $n \uparrow\infty$. For $i \in \mathbb {N}$, let

8.14 $$ A_{i} = \bigcup_{j = 1}^{i} E_{j} \subset B_{i}. $$

Then, for each $i \in \mathbb {N}$, $\hat{f}_{n}^{+}$ converges to $(f^{*})^{+}$ uniformly on $A_{i}$ as $n \uparrow\infty$. Thus (8.2) holds by Lemma 8.3.

Note that $\{A_{i}\}_{i \in \mathbb {N}}$ satisfies (2.1) and (3.2) by construction. Thus by Lemma 8.4, $\{A_{i}\} $ is a *σ*-finite exhausting sequence. Hence (3.1) holds by the hypothesis of Theorem 3.1. Since (8.2) also holds as shown in the previous paragraph, the Fatou inequality (1.1) holds by Lemma 8.2.

## Conclusions

In this paper we have provided a sufficient condition for what we call the Fatou inequality:

$$ \mathop {\overline {\lim }}_{n \uparrow\infty} \int f_{n} \,d\mu\leq \int \mathop {\overline {\lim }}_{n \uparrow\infty} f_{n} \,d\mu. $$

Our condition is considerably weaker than sufficient conditions known in the literature such as uniform integrability (in the case of a finite measure) and equi-integrability. We have illustrated the strength of our condition with simple examples. As an application, we have shown a new result on the existence of an optimal path for deterministic infinite-horizon optimization problems in discrete time. We have illustrated the strength of this existence result with concrete examples of optimization problems.
